# Supplementary material for: Frequency tagging of spatial attention using periliminal flickers
Source: Imaging Neurosci (Camb). 2024 Jul 12;2:imag-2-00223. doi: 10.1162/imag_a_00223 (PMC12272217; doi:10.1162/imag_a_00223)
Supplement: Supplementary Material [file imag_a_00223-supp.pdf]

1. Supplementary material

1.1. Time-frequency analysis of EEG data recorded over the occipital electrode Oz

In complement to the extraction of spectral features of SSVEP responses using the RESS methodology, this section present sensor-level event-related spectral perturbations (ERSP, Makeig (1993)) and Inter-Trial Coherence (ITC) measures. Although no statistical analyses were performed on these features, these dynamics are nevertheless presented in Supplementary Figure 1 to provide a comparative reference to the analyses performed on SSVEP SNR features extracted through the RESS method. Notably, the ITC provides a measure of SSVEP response consistency across trials. This measure complements the extraction of the SSVEP response SNR by providing a metric to assess how reliably and consistently SSVEP responses were evoked across single trials (Van Diepen and Mazaheri, 2018), providing valuable insight into understanding classification performance achieved across experimental conditions.

The computation of ERSP and ITC involved a time–frequency decomposition of the epoched data recorded over the midline occipital electrode Oz (over which SSVEP response is most prominent, Zheng et al. (2020)) through the convolution of complex Morlet wavelets. The number of wavelet cycles ranged from 3 to 32 following a 0.8-step increase to estimate frequencies ranging from 3 to 40 Hz in 74 linearly spaced frequency steps. The spectral power at each frequency was baseline-corrected using a decibel (dB) transform for each time point of the epoched data relative to the mean spectral activity recorded during the inter-trial interval (3 to 0 seconds relative to fixation phase onset) on a single-trial basis (Grandchamp and Delorme, 2011). The ERSP and ITC was computed for each point of the time-frequency array and averaged across trials.

The grand average (N=24) spectral perturbations and inter-trial coherence recorded over the midline occipital electrode ’Oz’ (where SSVEP responses are most prominent, Vialatte et al. (2010)) across experimental conditions are presented in 1. Akin to the reported RESS SNR, the ERSP measure is a measure of the amplitude/magnitude of the SSVEP response over frequencies and time. It is therefore without surprise that the ESRP recorded over cueing and target phases of the trials follow a similar pattern than the RESS SNR across conditions, with maximal response for the control condition (dark grey background), to reduced response for the periliminal flickers (light grey background), and minimal to seemingly absent SSVEP in response to subliminal flickers (transparent background). Interestingly, the ERSP plots also indicate the presence of a strong SSVEP response at the harmonic frequencies of both the 13 and 15 Hz flickers presented at the control condition intensity. The ITC plots reveal the presence of phase-locking responses across single trials over all three conditions, although this coherence appears to decrease as flicker intensity is reduced. This reduction in ITC, especially between periliminal and subliminal flicker intensities, may explain the differences observed in classification performance between the two conditions. Indeed, the subliminal stimuli appear to have induced phase-locked spectral responses less consistently from trial to trial. These inter-trial inconsistencies coupled with an overall reduced SSVEP response amplitude may explain the low classification accuracy achieved using subliminal flicker data.

1.2. Effects of flickers stimulation on endogenous alpha activity

To investigate the effect of visual stimulation on endogenous alpha power, we have computed the absolute power (through a Fourier transformation) of the fixation phase to compare it to the spectral power recorded during the cued phase. The rationale is that during the fixation phase, participants do not fixate or attend to the frequency-tagged area, while during the cueing phase, they both fixate and attend to the space where the flicker is present. This analysis was computed for each of the three conditions of amplitude depth. The reasoning here is that if our flicker had entrained endogenous alpha, this effect should be stronger in the control condition compared to the periliminal ones. We then ran a repeated-measures ANOVA with trial phase (fixation, cue), flicker frequency (13, 15Hz), and flicker intensity (control, periliminal, subliminal). The analyses revealed a main effect of the phase of the trial on mean alpha power [F(1,23) = 93.754, $p < .001$, $\eta_p^2 = = .803$]. There was no main effect of neither the flicker intensity [F(2,46) = 0.401, $p > .05$, $\eta_p^2 = = .017$] nor frequency [F(1,23) = 1.285, $p > .05$, $\eta_p^2 = = .053$]. The post-hoc tests revealed that mean absolute alpha power decreased during the cueing phase compared to the fixation phase ($p < .001$, d = 1.092). These results suggest that a suppression of alpha activity occurred when participants attended the frequency-tagged area in which the target is about to appear. This attentional effect has been described in previous work (Clements et al., 2023) The absence of a main effect of flicker frequency and of an interaction between the phase of the trial and flicker frequency on alpha band power suggests that attending to the 13 or 15 Hz flickers did not alter mean alpha absolute power. Furthermore,

the absence of main effects and interactions related to the flickers' intensity suggests that alpha power was not affected by the exogenous stimuli. While this analysis provides some insight into the reviewer's comment, it does not allow us to fully determine whether endogenous alpha would have affected the 13Hz and 15Hz SNR.

References

Clements, G.M., Gyurkovics, M., Low, K.A., Kramer, A.F., Beck, D.M., Fabiani, M., Gratton, G., 2023. Dynamics of alpha suppression index both modality specific and general attention processes. NeuroImage doi:10.1016/j.neuroimage.2023.119956.

Grandchamp, R., Delorme, A., 2011. Single-trial normalization for event-related spectral decomposition reduces sensitivity to noisy trials. Frontiers in Psychology 2, 1–14. doi:10.3389/fpsyg.2011.00236.

Makeig, S., 1993. Auditory event-related dynamics of the EEG spectrum and effects of exposure to tones. Electroencephalography and Clinical Neurophysiology 86, 283–293. doi:10.1016/0013-4694(93)90110-H.

Van Diepen, R.M., Mazaheri, A., 2018. The Caveats of observing Inter-Trial Phase-Coherence in Cognitive Neuroscience. Scientific Reports 8, 1–9. URL: http://dx.doi.org/10.1038/s41598-018-20423-z, doi:10.1038/s41598-018-20423-z.

Vialatte, F.B., Maurice, M., Dauwels, J., Cichocki, A., 2010. Steady-state visually evoked potentials: Focus on essential paradigms and future perspectives. Progress in Neurobiology 90, 418–438. doi:10.1016/j.pneurobio.2009.11.005.

Zheng, X., Xu, G., Wu, Y., Wang, Y., Du, C., Wu, Y., Zhang, S., Han, C., 2020. Comparison of the performance of six stimulus paradigms in visual acuity assessment based on steady-state visual evoked potentials. Documenta Ophthalmologica 141, 237–251. URL: https://doi.org/10.1007/s10633-020-09768-x, doi:10.1007/s10633-020-09768-x.

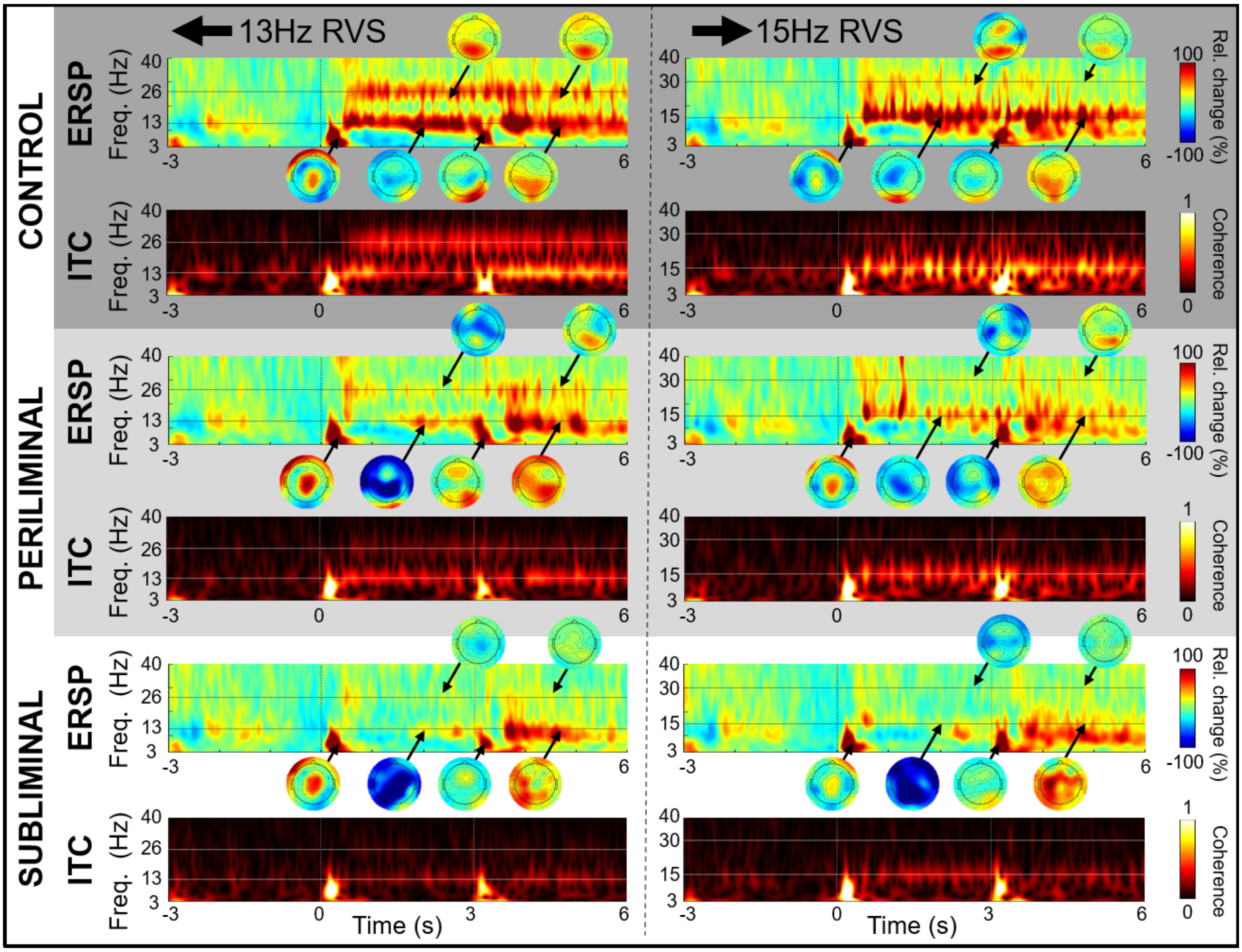

Figure 1: Grand average (N=24) spectral dynamics recorded over the occipital electrode 'Oz' for left (13Hz) and right (15Hz) flickers across each experimental condition (presented in the color-coded following panels). The Top-dark grey background: full amplitude depth modulation flickers, the middle-light grey background: periliminal flickers, bottom-white background: subliminal flickers. In each panel, the top plots present the Event-Related Spectral Perturbations (ERSP) expressed in percentage change from baseline (-1000 to 0ms before cue onset). Scalp maps reveal the spatial distribution of main modulations at frequency and times indicated by the arrows. The bottom plots highlight the Inter-Trial Coherence (ITC) of these spectral dynamics.
